# The impact of infectious diseases department on the incidence of hospital-onset bacteremia and fungemia at a tertiary care center: a retrospective cohort study

**DOI:** 10.1017/ice.2025.14

**Published:** 2025-04

**Authors:** Yuya Kawamoto, Akane Takamatsu, Kenjiro Matsui, Yohei Doi, Hitoshi Honda

**Affiliations:** 1 Department of Infectious Diseases, Fujita Health University School of Medicine, Toyoake, Aichi, Japan; 2 Department of Microbiology, Fujita Health University School of Medicine, Toyoake, Aichi, Japan; 3 Graduate School of Public Health, St. Luke’s International University, Chuo, Tokyo, Japan; 4 Department of Clinical Laboratory, Fujita Health University School of Medicine, Toyoake, Aichi, Japan; 5 Division of Infectious Diseases, University of Pittsburgh School of Medicine, Pittsburgh, Pennsylvania, United States; 6 Center for Infectious Diseases Research, Fujita Health University, Toyoake, Aichi, Japan; 7 Division of Infection Control, Department of Quality and Safety in Healthcare, Fujita Health University Hospital, Toyoake, Aichi, Japan

## Abstract

**Introduction:**

Cases of hospital-onset bacteremia and fungemia (HOBF) are on the rise in Japanese hospitals, but little is known about their incidence in hospitals and how it relates to the availability of services provided by infectious diseases departments.

**Methods:**

We herein investigated the monthly incidence density of HOBF per 1,000 patient days from 2013 through 2023 at a tertiary care hospital in Japan. The incidence of overall HOBF and pathogen-specific HOBF, including those caused by Enterobacterales, *Staphylococcus aureus*, coagulase-negative staphylococci (CNS), and *Candida* species, was tracked. Changes in the HOBF trend before and after the establishment of an infectious diseases department at the hospital were evaluated.

**Results:**

In total, 4,315 HOBF-related events were identified. The overall incidence density of HOBF increased by 2.4-fold from 0.58 per 1,000 PD in 2013 to 1.42 per 1,000 PD in 2023. Both the level and trend changes in the incidence density of overall HOBF (+0.3142 for change in level [*P* < .001]; +0.0085 for change in trend [*P* < .001]), HOBF caused by *S. aureus* (+0.0983 for change in level [*P* < .001]; +0.0016 for change in trend [*P* = 0.016]), and *Candida* spp. (+0.0466 for change in level [*P* = 0.030]; +0.0019 for change in trend [*P* = 0.002]) significantly increased after the establishment of the infectious diseases department.

**Conclusion:**

The incidence density of overall HOBF and clinically important pathogen-specific HOBF increased over the last decade. The availability of services through the infectious diseases department was significantly associated with an increase in the HOBF incidence, likely suggesting improvement in the diagnosis of HOBF.

## Introduction

Hospital-onset bacteremia and fungemia (HOBF) is one of the most critical infections that occur in the healthcare setting. A systematic review of HOBF surveillance systems estimated that 113,000-134,000 HOBF-related events occurred annually in North America, and 243,000-415,000 in Europe, respectively.^1^ Moreover, HOBF has been associated with increased mortality, prolonged hospitalization, and increased healthcare costs among inpatients.^2–4^ While the incidence density of central line-associated bloodstream infections (CLABSI) has conventionally been used as a cardinal metric for BSI surveillance due to hospital-onset (HO)-bacteremia commonly occurring in patients with a central venous catheter (CVC), this method may not be adequate in assessing the overall burden of BSI given the occurrence of HO-BSI in patients without a CVC.^5^ Among the objections to CLABSI incidence surveillance raised by some previous studies are its inability to accurately identify CLABSI cases, its reliance on the arbitrary determination of CLABSI by observers, and the difficulty of comparing the CLABSI incidence across hospitals.^6,7^ Given these limitations, the HOBF incidence, defined as BSI onset after three days of hospitalization, may be a better outcome measure to estimate the healthcare-associated infection burden, especially in acute care hospitals.^6,8^ Recent studies on the local epidemiology of HOBF, the proportion of causative pathogens, and changes in the HOBF trend have reported factors associated with differences in the proportion of causative pathogens in HOBF as well as regional differences and factors affecting its incidence.^9–14^

The infectious diseases (ID) subspecialty has gradually gained recognition over the last decade in the Japanese healthcare system, which typically provides ID clinical service and ID-related services, such as infection prevention and control (IPC) and antimicrobial stewardship programs (ASP). We hypothesized that establishment of ID services contributes to improved identification of HOBF cases in this setting. To test this hypothesis, we aimed to evaluate the long-term trend in the HOBF incidence at a single Japanese hospital where an ID department was newly established.

## Methods

### Study design and setting

The present retrospective, observational study was conducted at Fujita Health University Hospital (FHUH), a 1376-bed tertiary care university hospital in central Japan. Data on HOBF were collected from July 2013 through June 2023. During the study period, the incidence density of overall and pathogen-specific HOBF was tracked. The ID department at the study hospital was established in April 2018 and was followed immediately by the implementation of a bedside ID consultation service. A hospital epidemiologist and an ID physician from the department also supervised the IPC department and ASP, respectively. To test the aforementioned hypothesis of the study, changes in the HOBF incidence before and after the establishment of the ID department were assessed.

### Definition and study population

HOBF was defined as the onset of bacteremia and fungemia occurring after three calendar days of hospitalization at the study hospital.^8,15^ The diagnostic criterion for bacteremia and fungemia was one or more blood cultures with positivity for a known pathogen. For pathogens constituting the normal skin flora and potential contaminants (e.g., coagulase-negative staphylococci [CNS]), at least two sets of positive blood culture drawn on the same day were required for the diagnosis of true bacteremia. In cases of polymicrobial bacteremia, each pathogen was counted separately. HOBF was then further classified by causative pathogens, including Enterobacterales, lactose-non-fermenting gram-negative bacilli (e.g., *Pseudomonas* spp.), *Staphylococcus aureus*, CNS, streptococci, enterococci, and *Candida* species.

The incidence density of HOBF was calculated as the number of HOBF cases per 1,000 patient-days (PD), with the numerator being the number of cases per month and the denominator being the monthly total patient-days. The testing density of blood cultures, calculated as the number of blood cultures drawn per 1,000 PD with the number of blood cultures drawn per month as the numerator and the monthly total patient days as the denominator, was also tracked.

### Data collection

Data on HOBF were initially collected by the clinical microbiology laboratory at the hospital, included patient identifier, admission status, organism(s) isolated from blood cultures, date of the blood cultures, and the date of admission. Because follow-up blood cultures were commonly collected for HOBF due to *S. aureus* and *Candida* species, repeat blood cultures that grew these pathogens within three days from the first positive blood culture dates were excluded.^16^ In addition, one investigator (Y.K.) reviewed the electronic health records (EHR) of patients with a positive blood culture result for species present in the normal skin flora to determine the eligibility of such patients. The monthly numbers of positive blood culture results were extracted from a data warehouse in the EHR, and the monthly patient days were obtained from the IPC department. The monthly numbers of ID consultations after the introduction of ID clinical service were also tracked. Furthermore, the full-time equivalents of the ID clinical service, IPC department, and ASP were tracked.

### Statistical analysis

Interrupted time series analysis (ITSA) was conducted to evaluate the trend changes in HOBF before (July 2013 to March 2018) and after (April 2018 to June 2023) the establishment of the ID department using 120 data points at monthly intervals for each species and overall. The Newey-West method was used to fit the model, and the Cumby-Huizinga autocorrelation test was conducted.^17^ Seasonality was assessed to adjust its effect on the HOBF incidence, by incorporating a binary variable into the model, with a value of 1 assigned to the winter months (December, January, and February).^18^ This variable was included in the model only if it demonstrated statistical significance which was defined with *P* values < 0.05. In the subsequent ITSA model, *P* values < 0.05 were also considered to indicate statistical significance. All analyses were performed using Stata version 18 (StataCorp, College Station, TX, USA). The study was approved by the institutional review committee at FHUH (#HM23-280).

## Results

**Figure 1.**
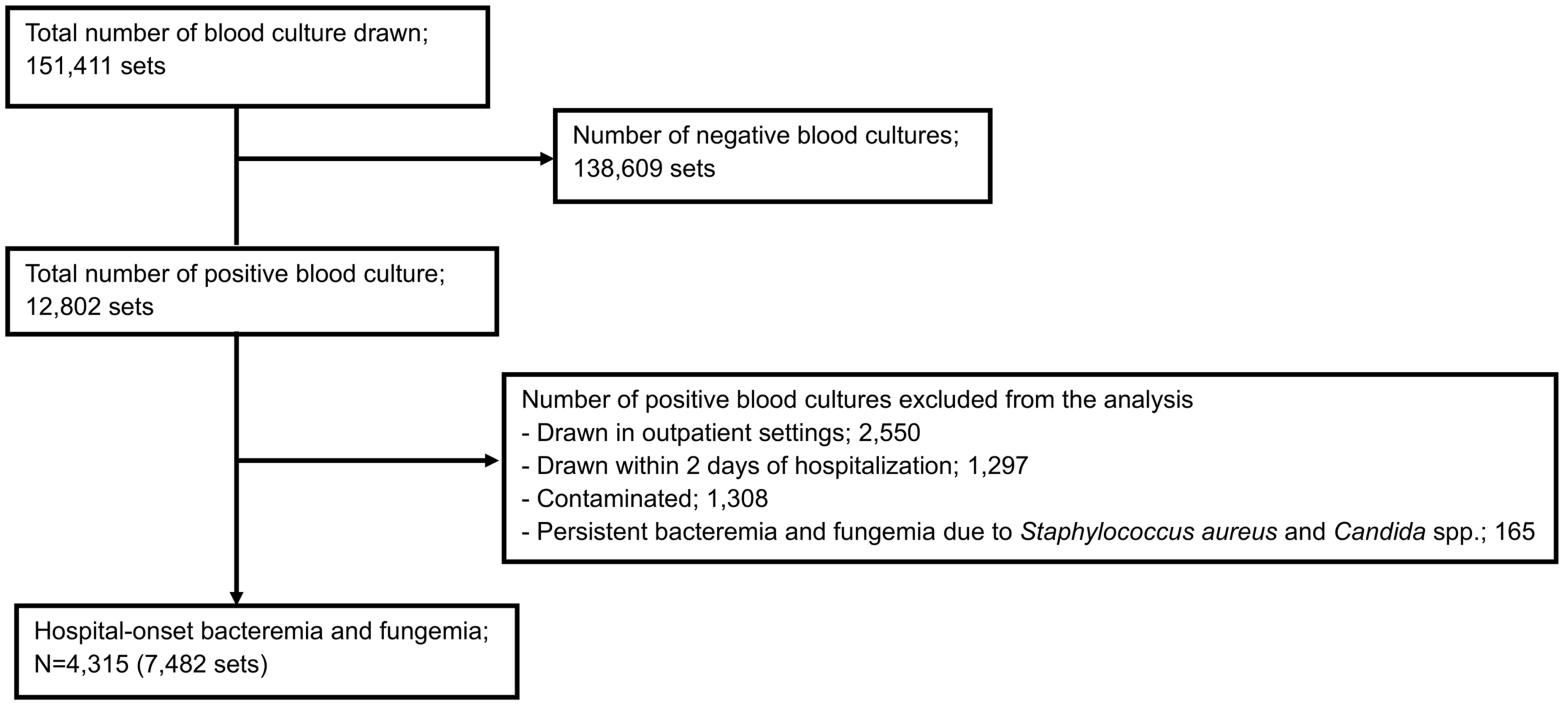
Distribution of blood cultures, patients with blood cultures, and cases of hospital-onset bacteremia and fungemia between 2013 and 2023.

During the study period, a total of 151,411 blood cultures were collected from 38,810 patients. Of these, 12,802 sets (8.5%) were positive. Of the positive blood cultures, 2,550 sets had been collected in outpatient settings, 1,297 sets had been collected within two days after admission, 165 sets were excluded due to persistent HOBF, and 1,308 sets were excluded as they represented contamination, leaving 7,482 positive blood cultures from 4,315 HOBF cases in 2,136 patients in the final analysis (Figure 1). Prior to 2018, ID clinical service was not available at the study hospital. Upon the establishment of the ID department, six board-certified ID specialists joined the study hospital as full-time faculty and launched an ID clinical service. A non-ID physician oversaw the IPC department prior to 2018. From 2018, one ID physician participated in the IPC department. In 2022, a hospital epidemiologist assumed the leadership of the IPC department. In addition, the number of designated infection control nurses in the IPC department increased from 2 to 5 in 2023.

ASP was established from the ground up upon founding of the ID department in 2018, and two ID physicians have actively been engaged in stewardship activities. One of the main ASP activities at the study hospital is prospective audit and feedback on antimicrobial therapy for patients with positive blood cultures, including HOBF.

The testing density of the monthly blood culture rates including all blood cultures drawn from both inpatients and outpatients at the hospital are shown in Supplementary Figure 1. Supplementary Figure 2 shows the trend of the monthly numbers of infectious diseases consultations.

The pathogens identified in the HOBF cases during the study period were the following in the order of decreasing proportions: CNS (n = 1,041; 24.1%), Enterobacterales (n = 939; 21.8%), *S. aureus* (n = 827; 19.2%), and *Candida* spp. (n = 475; 11.0%). Lactose-non-fermenting gram-negative bacilli and enterococci, which were less common, comprised 324 (7.5%) and 314 (7.3%) cases, respectively (Supplementary Table 1).

**Figure 2.**
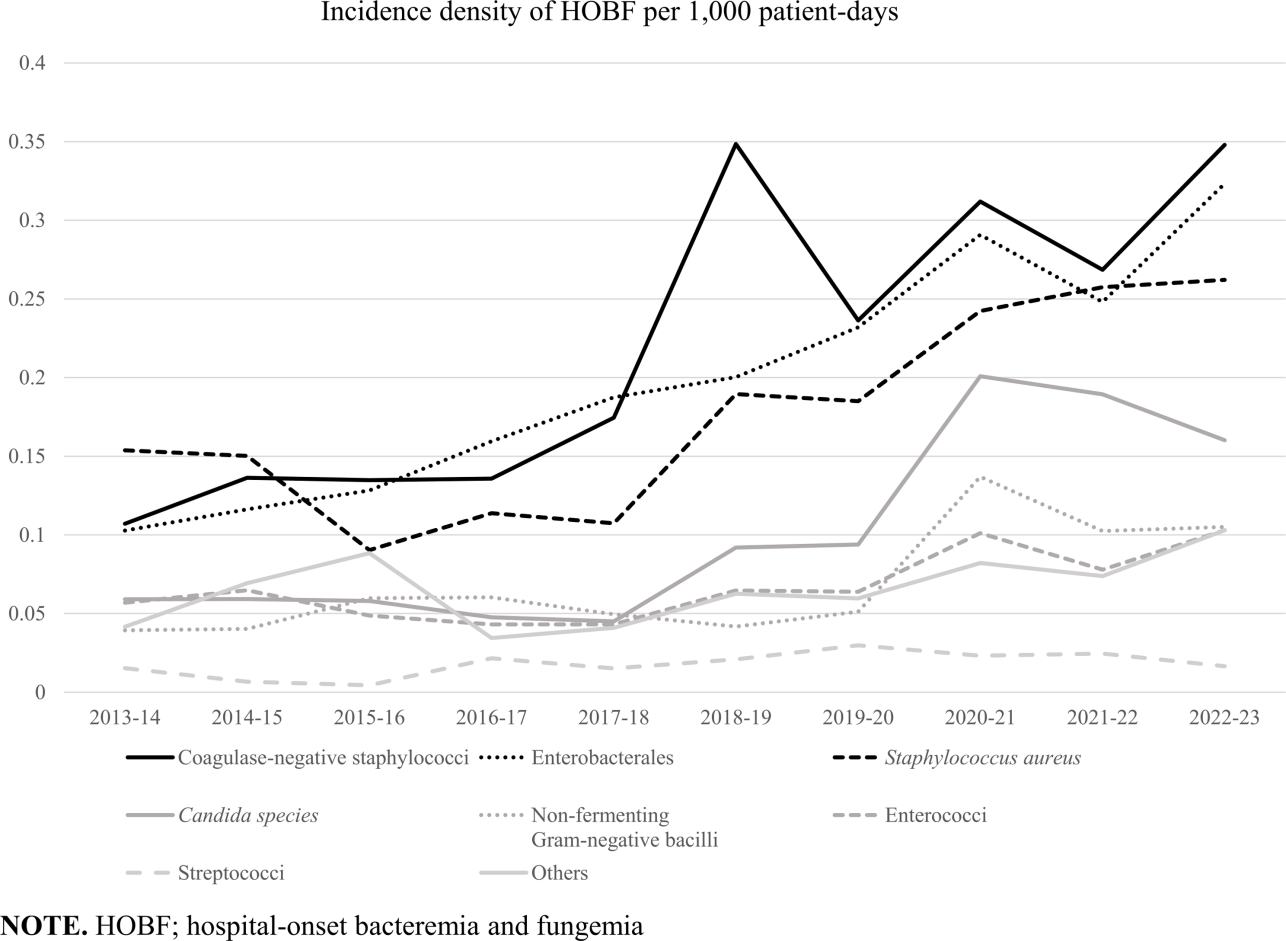
Trend in the annual incidence density of hospital-onset bacteremia and fungemia.

The overall incidence density of HOBF increased 2.4-fold between the first and the last years of the study period, from 0.58 per 1,000 PD in 2013 to 1.42 per 1,000 PD in 2023 (Table 1). A significant increase was observed in the annual HOBF incidence due to CNS (3.4-fold from 0.11 to 0.35 per 1,000 PD), Enterobacterales (3.1-fold from 0.10 to 0.32 per 1,000 PD), *S. aureus* (1.7-fold from 0.15 to 0.26 per 1,000 PD), *Candida* spp. (2.7-fold from 0.06 to 0.16 per 1,000 PD), enterococci (1.8-fold from 0.06 to 0.10 per 1,000 PD), and lactose-non-fermenting gram-negative bacilli (2.7-fold from 0.04 to 0.11 per 1,000 PD) (Figure 2).

**Table 1.** Annual incidence density of hospital-onset bacteremia and fungemia per 1,000 patient days from 2013 to 2023

Year^a^ Pathogen	2013-14	2014-15	2015-16	2016-17	2017-18	2018-19	2019-20	2020-21	2021-22	2022-23
All pathogens	0.577	0.641	0.613	0.614	0.663	1.020	0.947	1.396	1.244	1.419
Coagulase-negative staphylococci	0.107	0.136	0.135	0.136	0.175	0.349	0.236	0.312	0.269	0.348
Enterobacterales	0.103	0.116	0.128	0.159	0.187	0.200	0.232	0.291	0.248	0.323
*Staphylococcus aureus*	0.154	0.150	0.090	0.114	0.107	0.189	0.185	0.242	0.258	0.262
*Candida* species	0.059	0.059	0.058	0.048	0.045	0.092	0.094	0.201	0.189	0.160
Lactose non-fermenting gram-negative bacilli	0.039	0.040	0.060	0.060	0.050	0.042	0.051	0.137	0.103	0.105
Enterococci	0.057	0.065	0.049	0.043	0.043	0.065	0.064	0.101	0.078	0.103
Streptococci	0.015	0.007	0.004	0.022	0.015	0.021	0.030	0.023	0.025	0.017
Others	0.042	0.069	0.088	0.035	0.041	0.063	0.060	0.082	0.074	0.103

Note. Others included *Bacillus* species (spp)., *Corynebacterium* spp., *Lactobacillus* spp., and anaerobic bacteria (*Clostridium* spp., *Bacteroides* spp., *Peptostreptococcus* spp., *Fusobacterium* spp., *Prevotella* spp.).

a The study period started from July 2013 through June 2023.

**Figure 3.**
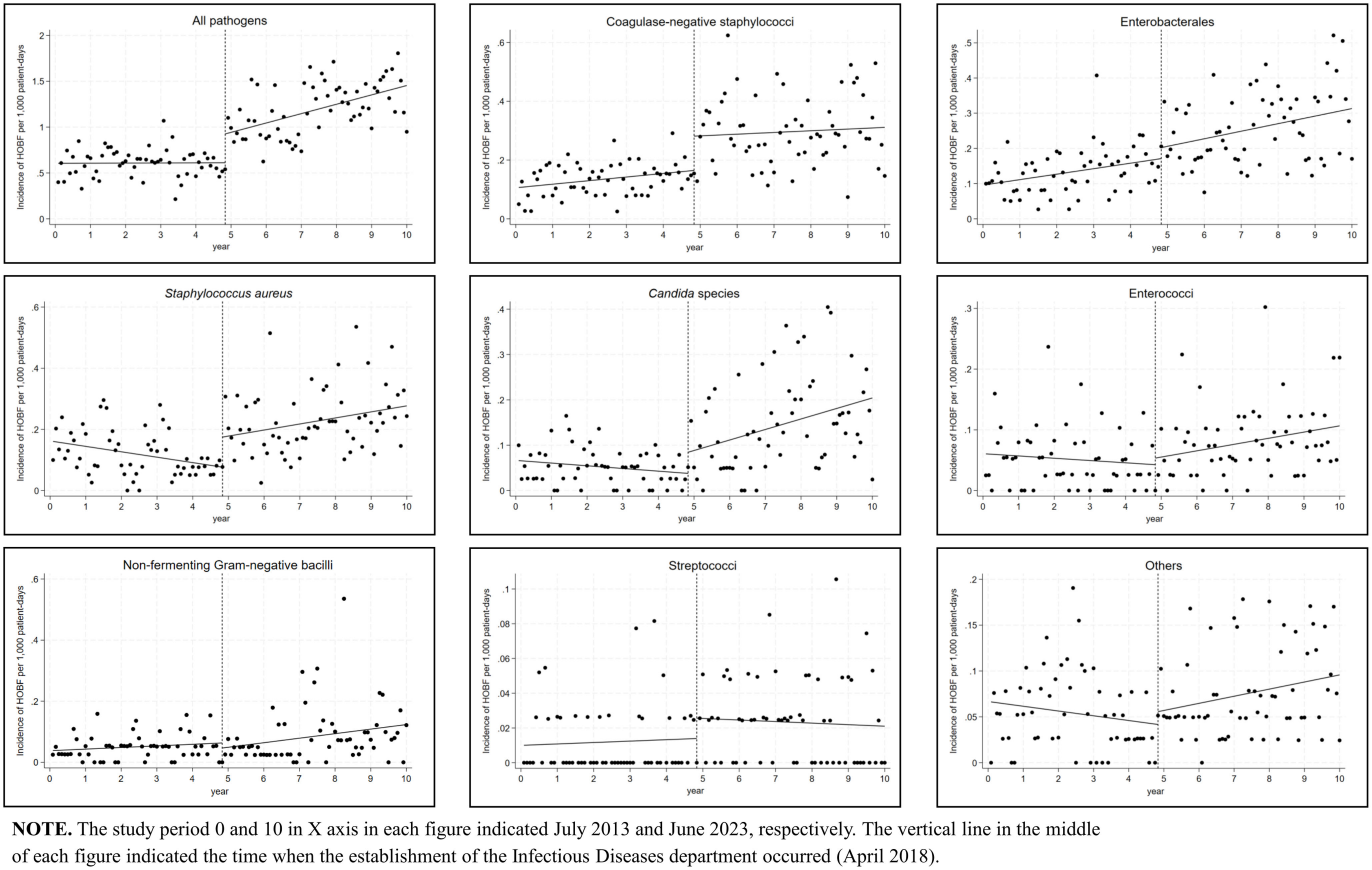
Changes in the incidence density of hospital-onset bacteremia and fungemia before and after the establishment of the infectious diseases department.

The incidence density of the overall HOBF increased significantly after the establishment of the ID department based on the ITSA (+0.3142 for change in level [*P* < .001]; +0.0085 for change in trend [*P* < .001]) (Table 2 and Figure 3). Moreover, significant increase was observed for both the level and trend changes in the HOBF incidence density due to *S. aureus* (+0.0983 for change in level [*P* = 0.001]; +0.0016 for change in trend [*P* = 0.016]), and *Candida* spp. (+0.0466 for change in level [*P* = 0.030]; +0.0019 for change in trend [*P* = 0.002]). The incidence density of HOB caused by enterococci, Enterobacterales, and lactose-non-fermenting gram-negative bacilli also revealed a significant upward trend following the establishment of the ID department (+0.0009 [*P* = 0.016]; +0.0018 [*P* = 0.014]; +0.0012 [*P* = 0.005] for change in trend, respectively), whereas the change in level was not significant for these species (+0.0109 [*P* = 0.446]; +0.3138 [*P* = 0.242]; -0.0166 [*P* = 0.334] for change in level, respectively). An immediate and significant increase in the incidence density of HOB caused by CNS was observed (+0.1167 for change in level [*P* = 0.001]) whereas the upward trend in the incidence did not reach statistical significance (+0.0005 for change in trend [*P* = 0.58]).

**Table 2.** Assessment of changes in the incidence density of hospital-onset bacteremia and fungemia before and after the establishment of the infectious diseases department using interrupted time series analysis

Incidence of HOBF per 1000 patient days								
	Trend before the establishment of ID department (95% CI)	*P* value	Change in level (95% CI)	*P* value	Trend after the establishment of ID department (95% CI)	*P* value	Change in trend (95% CI)	*P* value
All pathogens	+0.0001 (–0.0021, +0.0023)	0.927	+0.3142 (+0.1678, +0.4607)	< 0.001	+0.0084 (+0.0043, +0.0125)	< 0.001	+0.0085 (+0.0051, +0.0120)	< 0.001
Coagulase-negative staphylococci	+0.0010 (–0.0002, +0.0018)	0.002	+0.1167 (+0.0504, +0.1830)	0.001	–0.0005 (–0.0024, +0.0014)	0.579	+0.0005 (–0.012, +0.0022)	0.580
Enterobacterales	+0.0013 (+0.0005, +0.0021)	0.002	+0.3138 (–0.0210, +0.8419)	0.242	+0.0005 (–0.0013, +0.0021)	0.553	+0.0018 (+0.0004, +0.0032)	0.014
*Staphylococcus aureus*	–0.0015 (–0.0023, –0.0006)	0.001	+0.0983 (+0.0439, +0.1526)	0.001	+0.0031 (+0.0015, +0.0047)	< 0.001	+0.0016 (+0.0003, +0.0030)	0.016
*Candida* species	–0.0005 (–0.0011, +0.0001)	0.085	+0.0466 (+0.0046, +0.0886)	0.030	+0.0024 (+0.0011, +0.0038)	< 0.001	+0.0019 (+0.0007, +0.0031)	0.002
Lactose non-fermenting gram-negative bacilli	+0.0004 (–0.0002, +0.0011)	0.172	–0.0166 (–0.0505, +0.1730)	0.334	+0.0008 (–0.0003, +0.0019)	0.137	+0.0012 (+0.0004, +0.0021)	0.005
Enterococci	–0.0003 (–0.0008, +0.0002)	0.195	+0.0109 (–0.1730, +0.3907)	0.446	+0.0012 (+0.0003, +0.0020)	0.006	+0.0009 (+0.0002, +0.0016)	0.016
Streptococci	+0.0001 (–0.0021, +0.0004)	0.659	+0.1170 (–0.0027, +0.0261)	0.110	–0.0001 (–0.0005, +0.0003)	0.499	+0.0001 (–0.0004, +0.0002)	0.610
Others^a^	–0.0004 (–0.0010, +0.0001)	0.116	+0.0140 (–0.0127, +0.0407)	0.301	+0.0011 (+0.0003, +0.0019)	0.010	+0.0006 (+0.0001, +0.0013)	0.037

Note. HOBF, hospital-onset bacteremia and fungemia; CI, confidence interval. ^a^Others included *Bacillus* species (spp.), *Corynebacterium* spp., *Lactobacillus* spp., and anaerobic bacteria (*Clostridium* spp., *Bacteroides* spp., *Peptostreptococcus* spp., *Fusobacterium* spp., *Prevotella* spp.)

## Discussion

The present analysis of 4,315 HOBF cases from a ten-year study period at a Japanese university hospital found that the incidence density of overall HOBF and most categories of pathogen-specific HOBF increased considerably. Moreover, the establishment of the ID department at the study hospital was significantly associated with an increase in the HOBF incidence, as confirmed by ITSA.

The reason behind the observed increase in the incidence density of overall HOBF was likely multifactorial. Previous studies reported that an increase in the number of inpatients, especially elderly patients, with a high HOBF risk combined with changes in clinical practice, such as the frequent use of intravascular devices, particularly in high-income countries, and improvements in microbiological techniques led to an increase in the HOBF incidence.^19–22^

The present study also highlighted significant increase in the HOBF incidence following the establishment of the ID department. The increase in both the immediate and trend levels was significant owing to the considerable number of pathogens implicated, as confirmed by ITSA. The establishment of the ID department at the study hospital led to the implementation of an ID clinical service and ASP, both of which substantially augmented the effectiveness and reach of the preexisting IPC department. Prior to establishment of the ID department, the number of blood cultures per 1,000 patient days was substantially lower than the number of blood cultures recommended by the Clinical and Laboratory Standards Institute, suggesting that blood culture practice prior to the introduction of ID services was likely suboptimal.^23^ However, as clinicians at the hospital gained experiences in managing patients with suspected infection, the number of blood cultures performed per 1,000 patient days gradually increased (Supplementary Figure 1). In fact, a collaborative partnership between the ID clinical service and ASP/IPC teams is an essential component in enhancing blood culture performance and utilization.^24^ Moreover, the numbers of ID consultations increased over time, which may have positively impacted hospital-wide ID practice and improved the detection of HOBF (Supplementary Figure 2). Although the growing number of vulnerable inpatients (e.g., elderly inpatients) may also account for the rise in the HOBF incidence, the positive association between the introduction of ID services and the HOBF incidence on both the immediate and trend levels likely reflects the effectiveness of the ID services provided at the study hospital.

A previous, nationwide surveillance study in the US, which included 24,179 HOB cases, revealed that CNS was the most prevalent pathogen, accounting for 31% of HOB cases, followed by *S. aureus*, which is consistent with the findings in the present study.^9^ Because staphylococci are the leading cause of CLABSI and other catheter-related BSI, it is likely that CLABSI is the most frequent cause of HOBF. However, recent studies in some Asian countries demonstrated that HOBF due to gram-negative bacilli has become predominant, with *Klebsiella* spp. being the most commonly identified pathogen and *S. aureus* accounting for only 2-9% of HOBF cases.^13,14^ Differences in patient characteristics, such as age, hospital ward at HOBF onset, and length of stay, may lead to differences in the prevalence of certain causative pathogens.^9,14,25^ Furthermore, previous studies suggested that higher temperatures might be associated with an increased incidence of HOB due to gram-negative bacilli.^26–28^ The concerns over the potential impact of climate change on healthcare outcomes also support the need to track the long-term epidemiology of HOBF.

We acknowledge several limitations of the study. Although persistent HOBF due to *S. aureus* and *Candida* spp. were excluded from the analysis, duplication may have occurred if patients had persistent bacteremia or fungemia from other less common pathogens. The impact of the COVID-19 pandemic on the HOBF incidence was not assessed whereas some studies have reported a temporary increase in the CLABSI incidence during the pandemic.^29,30^ However, the proportion of inpatients with COVID-19 was much smaller than that of inpatients without COVID-19 even at the height of the pandemic, with the highest proportion of COVID-19-related hospitalization of approximately 4.7% (data not shown). Thus, the effect of COVID-19 on the overall HOBF incidence over the 10-year period is likely modest. The epidemiological data derived from the hospital EHR warehouse did not take into account patient-related factors, such as their demographic and clinical characteristics, which may explain at least some aspects of the differences in the HOBF incidence. For instance, a previous study revealed that HOBF incidence may be influenced by the patients’ age.^20^ In-depth, patient-level examination of clinical and healthcare outcomes of HOBF would require a different study design and will be our next step in the investigation. Furthermore, the blood culture practice prior to the introduction of ID department was likely inadequate, suggesting that HOBF patients had been underdiagnosed; however, as this study did not collect individual patient data, the this possibility could not be conclusively confirmed. Lastly, since the present study did not assess changes in the infectious diseases-related practice pattern at the patient level after the introduction of the ID services, actual changes in practice could not be elucidated. However, the results obtained from long-term observational data using ITSA and the increasing blood culture collection rates during the study period suggest that such improvement likely occurred. Although the association between the introduction of ID services and increased HOBF detection due to more frequent blood culture collection likely represents a process improvement around the ID clinical practice, monitoring for excessive blood culture collection or blood culture contamination should be prioritized and advocated, especially in the era of diagnostic stewardship.

In conclusion, the HOBF incidence substantially increased during a recent 10-year period at the study hospital. Tracking the HOBF incidence can help us better understand the overall healthcare burden associated with bacteremia and fungemia in the acute healthcare setting. Moreover, the availability of comprehensive ID services, including consultation, ASP and IPC, may contribute to improving the diagnosis of HOBF while optimizing blood culture collection practice both in short and long terms, which underscores the pivotal role of these services in improving patient care. Given the substantial morbidity and mortality related to HOBF, continued effort is needed to develop a strategy for identifying HOBF cases effectively.

## Supporting information

Kawamoto et al. supplementary material Kawamoto et al. supplementary material

## Data Availability

The data underlying this article will be shared on reasonable request to the corresponding author.

## Supplementary material

The supplementary material for this article can be found at https://doi.org/10.1017/ice.2025.14
