# Supplementary material for: The impact of infectious diseases department on the incidence of hospital-onset bacteremia and fungemia at a tertiary care center: a retrospective cohort study
Source: Infect Control Hosp Epidemiol. 2025 Feb 14;46(4):363–9. doi: 10.1017/ice.2025.14 (PMC12015624; doi:10.1017/ice.2025.14)
Supplement: Kawamoto et al. supplementary material [file S0899823X25000145sup001.docx]

**Appendix.** The impact of infectious diseases department on the incidence of hospital-onset bacteremia and fungemia at a tertiary care center: a retrospective cohort study

**Authors:** Yuya Kawamoto M.D^.^, Akane Takamatsu M.D., Ph.D., Kenjiro Matsui M.S., Yohei Doi M.D., Ph.D., Hitoshi Honda M.D., Ph.D.

**Supplementary Table 1.** The etiology of hospital-onset bacteremia and fungemia

| Pathogen (N=4,315) | The number (proportion, %) |
| --- | --- |
| Coagulase-negative staphylococci | 1,041 (24.1) |
| Enterobacterales | 939 (21.8) |
| *Staphylococcus. aureus* | 827 (19.2) |
| *Candida* species | 475 (11.0) |
| Non-fermenting Gram-negative bacilli | 324 (7.5) |
| Enterococci | 314 (7.3) |
| Streptococci | 84 (1.9) |
| Others | 311 (7.2) |

**NOTE.** Others included *Bacillus* species (spp.), *Corynebacterium* spp., *Lactobacillus* spp., anaerobic bacteria (*Clostridium* spp., *Bacteroides* spp., *Peptostreptococcus* spp., *Fusobacterium* spp., *Prevotella* spp.

**Supplementary Figure 1.** The trend of the testing density of the monthly number of blood cultures per 1,000 patient-days (all blood cultures drawn at the study hospital)


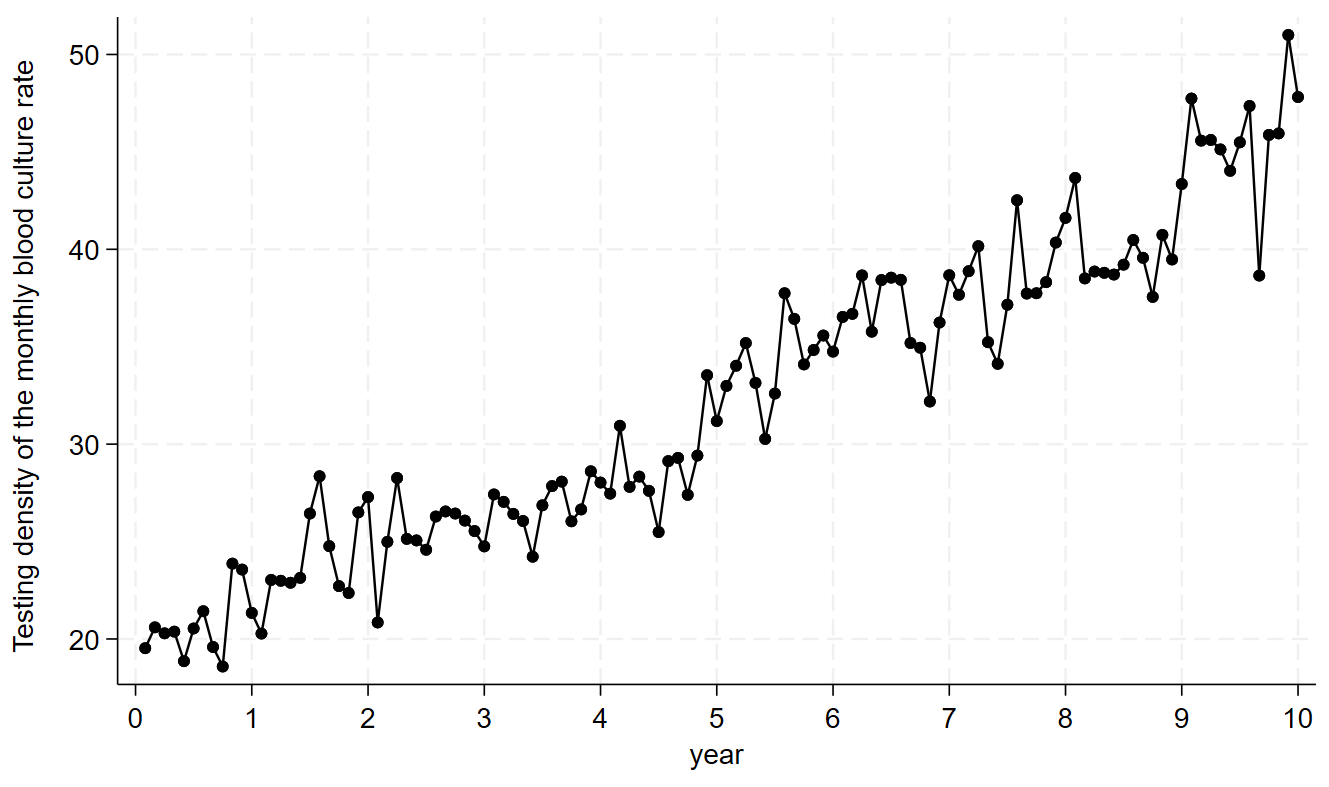


**NOTE.** The study period 0 and 10 in X axis in each figure indicated July 2013 and June 2023, respectively. Interrupted time series revealed that an increasing trend of blood culture testing density was associated with the establishment of the infectious diseases department (+2.501 for change in level [ P<.001], +0.2187 for change in trend [P<.001]).

**Supplementary Figure 2.** The trend of the monthly number of Infectious Diseases consultations


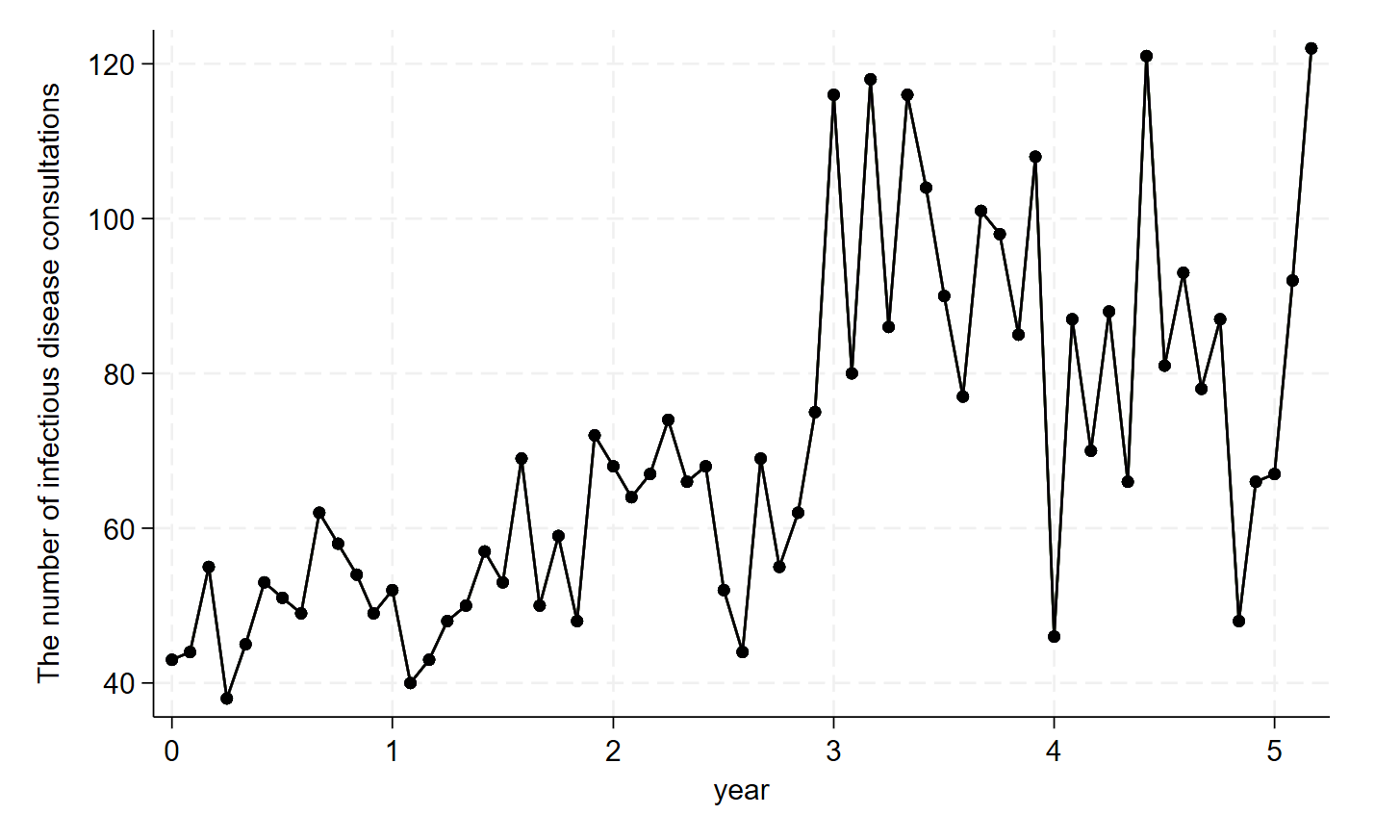
**NOTE.** The study period 0 and 5 in X axis in each figure indicated April 2018 and April 2023, respective.
